# An open-source LED array illumination system for automated multiwell plate cell culture photodynamic therapy experiments

**DOI:** 10.1038/s41598-022-22020-7

**Published:** 2022-11-11

**Authors:** Kai Zhang, Sudip Timilsina, Matthew Waguespack, Eric M. Kercher, Bryan Q. Spring

**Affiliations:** 1 Translational Biophotonics Cluster, Northeastern University, 360 Huntington Ave., Boston, 02115 USA; 2 Department of Physics, College of Science, Northeastern University, 360 Huntington Ave., Boston, 02115 USA; 3 University of Massachusetts Medical School, 55 Lake Ave. N, Worcester, MA 01655 USA; 4 Department of Bioengineering, College of Engineering, Northeastern University, 360 Huntington Ave., Boston, 02115 USA

**Keywords:** Cancer therapy, Biomedical engineering

## Abstract

Photodynamic therapy (PDT) research would benefit from an automated, low-cost, and easy-to-use cell culture light treatment setup capable of illuminating multiple well replicates within standard multiwell plate formats. We present an LED-array suitable for performing high-throughput cell culture PDT experiments. The setup features a water-cooling loop to keep the LED-array temperature nearly constant, thus stabilizing the output power and spectrum. The setup also features two custom-made actuator arms, in combination with a pulse-width-modulation (PWM) technique, to achieve programmable and automatic light exposures for PDT. The setup operates at ~ 690 nm (676–702 nm, spectral output full-width half-maximum) and the array module can be readily adapted to other LED wavelengths. This system provides an illumination field with adjustable irradiance up to 400 mW/cm^2^ with relatively high spectral and power stability comparing with previously reported LED-based setups. The light doses provided by the LED array were validated with comparison to traditional laser PDT. This open-source illumination platform (including the detailed technical description, fabrication protocols, and parts list provided here) helps to make custom light sources more accessible and of practical use for photomedicine research.

## Introduction

Photodynamic therapy (PDT) uses light of a specific wavelength to excite non-toxic photo-sensitive chemicals, usually referred to as photosensitizers, to generate cytotoxic reactive oxygen species (ROS)^1,2^. PDT provides a localized, controllable approach to provide treatment to a broad range of diseases, including various cancers^3,4^. As it provides unique mechanism to cell damage distinct from chemotherapy^5^ and can stimulate immunogenic cell death^6^, PDT is often effective in killing chemo-resistant cells, which opens opportunities to prime standard chemotherapy and immunotherapy for treating cancers^7,9^. For instance, PDT is in clinical trial of treating pancreatic cancer and head and neck cancer^10,11^.

Many kinds of light delivery systems for PDT have been developed to meet the requirements for different indications and scenarios. Lasers, with precise wavelength and stable output, are the most widely used sources^12–15^. The coherence of lasers makes them easily coupled with the optical fiber to reach inside the body for endoscopy-based light delivery^12,14^. In contrast, light-emitting diodes (LEDs) are non-coherent and do not achieve the energy density of coherent sources, but they can be combined into a low-cost array to increase the power output, which is efficient for wide-field clinical skin treatments^16^ and in vitro cell culture experiments for early-stage studies of photomedicine. LEDs have similar efficacy to lasers when the LED output spectrum overlaps strongly with the photosensitizer absorption spectrum^15,17–19^.

LED-based PDT setups must overcome several practical challenges. LEDs typically have a wide spectral bandwidth compared to lasers, which reduces the photosensitizer absorption efficiency per photon emitted compared to a laser, though this can be compensated by increasing the output power of the LED array. Furthermore, the heat generated by the high-power LEDs can not only shift the output spectrum, which further reduces the absorption efficiency, but also heat the target and create unwanted hyperthermic effects. In addition, the nonlinear current–voltage response adds difficulty to adjusting the output power without a closed-loop control system.

A variety of innovative LED illumination systems have been developed for in vivo PDT. Early in 1993, an LED-array-based source was developed with comparable power to sunlight^20^. Implanted LEDs that are energized remotely were designed to achieve deep tissue, controlled-dose, and low-power PDT^21,22^. Liu et al. developed a battery-operated, low-cost, and portable device that is useful in resource-limited environments^23^. However, there are only a few papers discussing the development of high-power in vitro setups that are useful for high-throughput cell culture tests necessary to discover novel photomedicines and PDT-based combination therapies. Note that commercial products that can provide high-quality illumination for scientific research are presently very rare. LED BOX^24^ (BioLambda; 50 mW/cm^2^, 90% uniformity) is a promising available product. However, this product does not offer programmable irradiance for multi-well plates as developed here.

Here, we present an open-source LED-array-based PDT setup, including hardware and software, designed for in vitro experiments that overcomes the challenges listed above and we anticipate that the information detailed here will enable PDT researchers to build for their own similar devices. The setup is based on our previous LED array design^25^, with several improvements. The new system features a modular design that eases customization of the LED type used and the operating wavelength, and a water-cooling loop has been integrated to dissipate heat and to stabilize the LED temperature and output spectrum. The new setup also features a pair of robot arms (actuators) to move the plate, enabling hand-free PDT experiments while the user only needs to program the dose for each well or experimental group of replicate wells. Finally, we demonstrate a pulse width modulation (PWM) technique as a convenient and linear method to control the LED array irradiance.

## Results

### Automated LED array design

**Figure 1:**
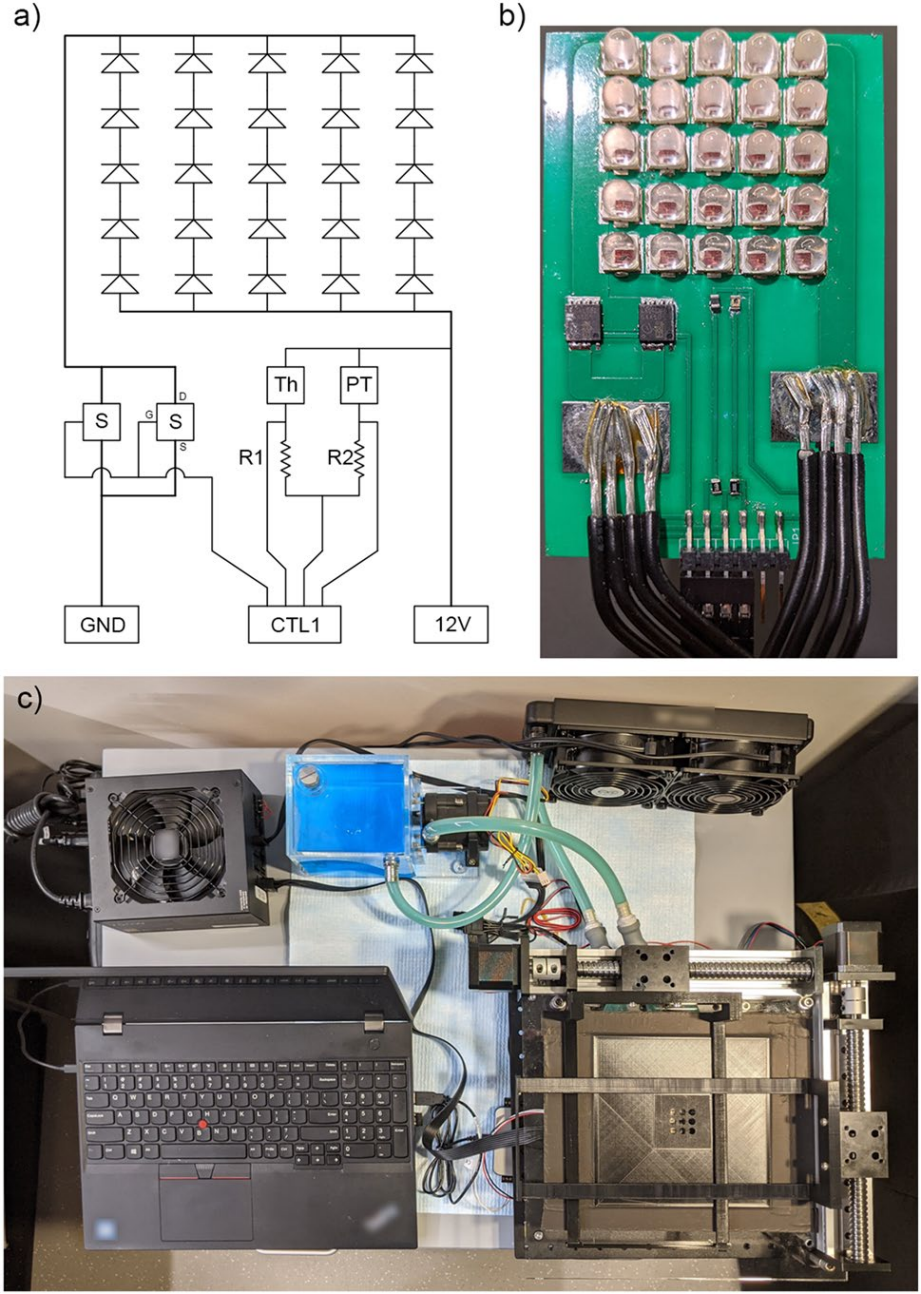
(**a**) A schematic top view of the LED array PCB. The positions of the components match the actual position on board. S, MOSFET switch; Th, thermistor; PT, phototransistor; CTL1, connector to the DAQ; R1 and R2, resistors. (**b**) Photograph of the PCB corresponding to the schematic in (**a**). Note that only 4 pins of the 6-pin connector are used. The extra two pins are for mechanical strength. (**c**) Photograph of the entire LED illumination system including a laptop and a water-cooling loop with fans. The LED array PCB is below the robotic arms and sample holder (bottom right corner).

The LED array design includes several auxiliary systems to support the uniformity and stability of light delivery, including the following major hardware components: a printed circuit board (PCB, Fig. 1a,b); optics that collimate the light to the sample well plate; a water-cooling system that keeps the LED array at constant temperature (Fig. 1c); two robot arms that move the plate to enable automation (Fig. 1c); a computer and a data acquisition (DAQ) board to control the above components; and, a power supply to provide 12 V DC. The LED array itself consists of 25 high-power LEDs (Fig. 1b). Groups of five LEDs are connected in series, and each of these serial branches are connected in parallel to form a 5-by-5 matrix. This array can generate an irradiance of 400 mW/cm^2^ for a 2-inch-diameter area in the sample plane. The PCB features a thermistor and a phototransistor to monitor the temperature and the light intensity of the LED array. The voltage divider circuit and the calibration process of the thermistor have been detailed previously^25^. The phototransistor circuit has a similar design as the thermistor circuit, and a linear fit of the output voltage versus the LED array light intensity may be used for calibration. Despite phototransistor saturation effects (detailed in the Rohm RPM-075PTT86 phototransistor data sheet), the fit agrees with the measured values with an error of less than 2 mW (Fig. 3a).

**Figure 2:**
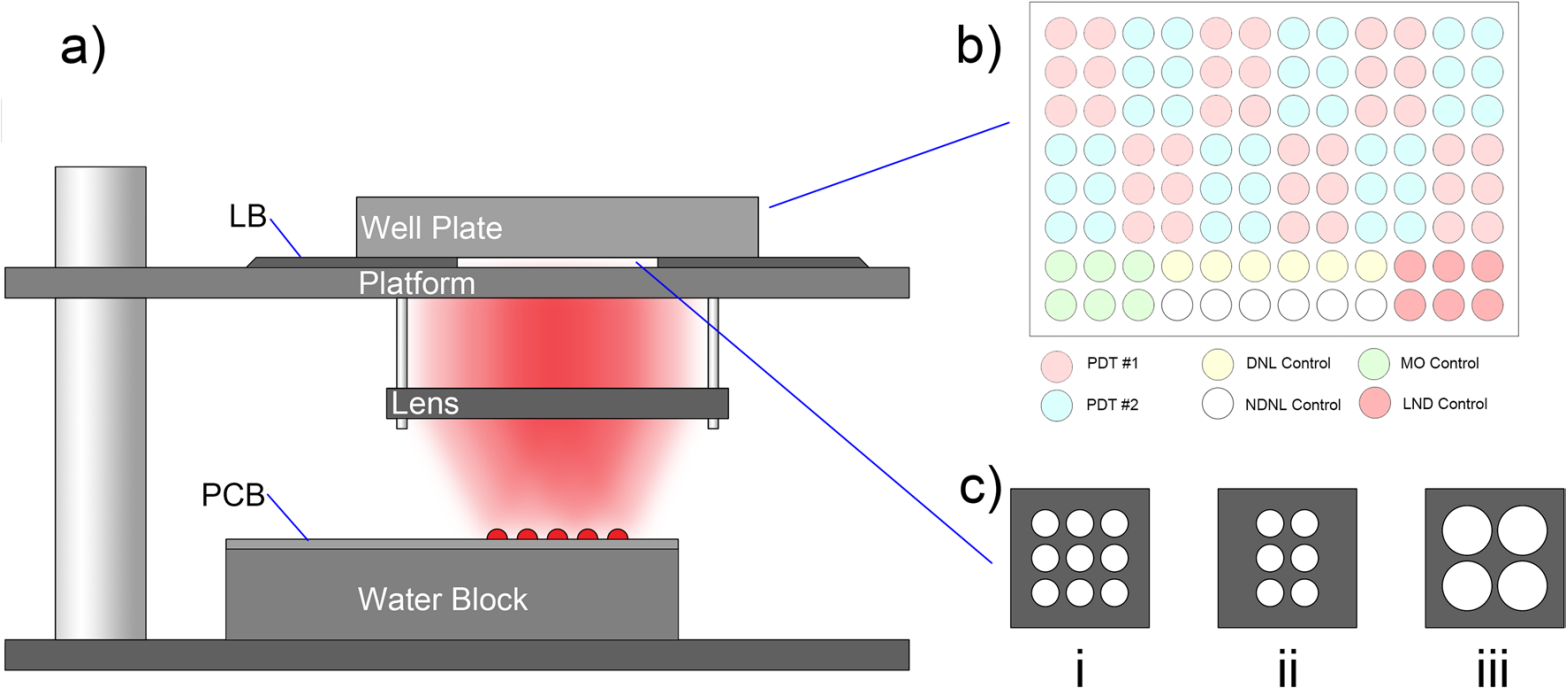
Optical setup for a 96-well plate. (**a**) A side view of the optical setup. LB, 3D-printed light blocker. (**b**) The well plate experimental group layout used for light dose response tests. PDT #1, PDT #2, two different treatment groups; DNL, drug with no light; NDNL, no drug with no light; MO, media only; LND, light with no drug. The staggered layout of PDT #1 and PDT #2 are suggested as a means to detect an incorrect alignment using the automated illumination system, which is an easy mistake in our experience when first setting up this system. Details regarding the staggered layout are described in the Supplementary Information *(Well Plate Layout)*. (**c**) The top view of the spatial light filter for (i) 9-well group for a 96-well plate, (ii) 6-well group for a 96 well plate, as depicted in (**b**), and (iii) 4-well group for a 24-well plate.

A Fresnel lens with a diffusing surface is placed above the LED array to help provide a relatively uniform and collimated distribution of light to the sample wells (Fig. 2a). To approximately match the size of the LED array, 2-inch optics are used. This approach enables uniform light dose application to 9 wells (a 3-by-3 matrix in a 96-well plate) simultaneously, which can be leveraged to shorten the duration of PDT experiments (Fig. 2b,c) as opposed to illumination one well at a time). Above the Fresnel lens, an acrylic board is mounted as the platform to hold the sample well plate (Fig. 2a). The board is covered with a black, 3D printed light blocker to block unwanted LED light outside of the perimeter of the well plate, with a transparent square region in the center through which the light can be transmitted. Beyond the edges of the light blocker, aluminum tape is used to cover the entire area of the platform. A 3D-printed spatial light filter (Fig. 2c) is placed in the center square region to further shape the light so that the light is only transmitted to the wells of interest. This spatial filter is critical for mitigating stray light absorbed by the black walls of well plates, which can otherwise lead to significant unwanted heating of the cell culture media (particularly for the small volumes used in 96-well plates). This two-part design can be easily modified to adapt to different well plate layouts while maintaining alignment. The well plate is covered by a 3D-printed cover with black, non-reflective tape to prevent the light from scattering to the adjacent wells from the top.

A typical in vitro PDT experiment requires multiple light doses and replicates of each light dose to generate a dose–response curve. This requires moving the plate to align the wells-of-interest to the transparent region and to make sure no light leaks into the adjacent wells. Manual plate positioning for each light exposure requires constant user interaction and concentration to avoid mistakes, which motivates developing an automated process. Here we introduce an automated system using two stepper-motor-based robot arms to move the sample well plate. Two 3D-printed rails hold the well plate in the x and y directions. The rails are mounted onto the screw actuators driven by the stepper motors. When the motor rotates, the corresponding rail moves the plate in one direction. The well plate is not fixed to the rails, so it is free to slide along the arms, making the movements in the two dimensions independent (Supplementary Video 1). The screw actuators provide a motion precision of 25 μm/step (5 mm pitch, 400 steps/rotation). The two stepper motors are controlled by the computer-controlled data acquisition board (DAQ). A custom-made, LabVIEW-based program (Supplementary Software) controls the robot arms to move the plate to the pre-determined position for each group of wells. The user only needs to enter the light dose to be applied to each group before the experiment, and then the setup will calculate the timing, move the well plate, and perform the PDT automatically (see GUI shown in the Supplementary Video).

To test whether the robot arms move the well plate with more precision than human hands, eliminating the light dose error from the randomness of the well plate position, we performed an experiment to move the well plate to a pre-determined location with repeated position measurements captured by a camera. The actual location distribution of the automated robot arms is an order of magnitude smaller than the manual, human results (FWHMs are 0.02 mm for the robot arms and 0.47 mm for manual human placements).

### PWM provides flexible irradiance control for PDT

The peak power of the LED array (400 mW/cm^2^) is much larger than the intensity used in a typical PDT experiment. While increasing the intensity reduces the time required to apply the desired light dose, some saturation effects, like the depletion of local molecular oxygen, could occur^26^. Therefore, it is necessary to reduce the output power of the LEDs. A typical method to control the power is to reduce the DC voltage supplied^27^. However, high-power variable power supplies are typically bulky and require more intricate integration than constant voltage supplies. In addition, the nonlinear I-V response of the LED makes it difficult to control the output power without a feedback system.

**Figure 3:**
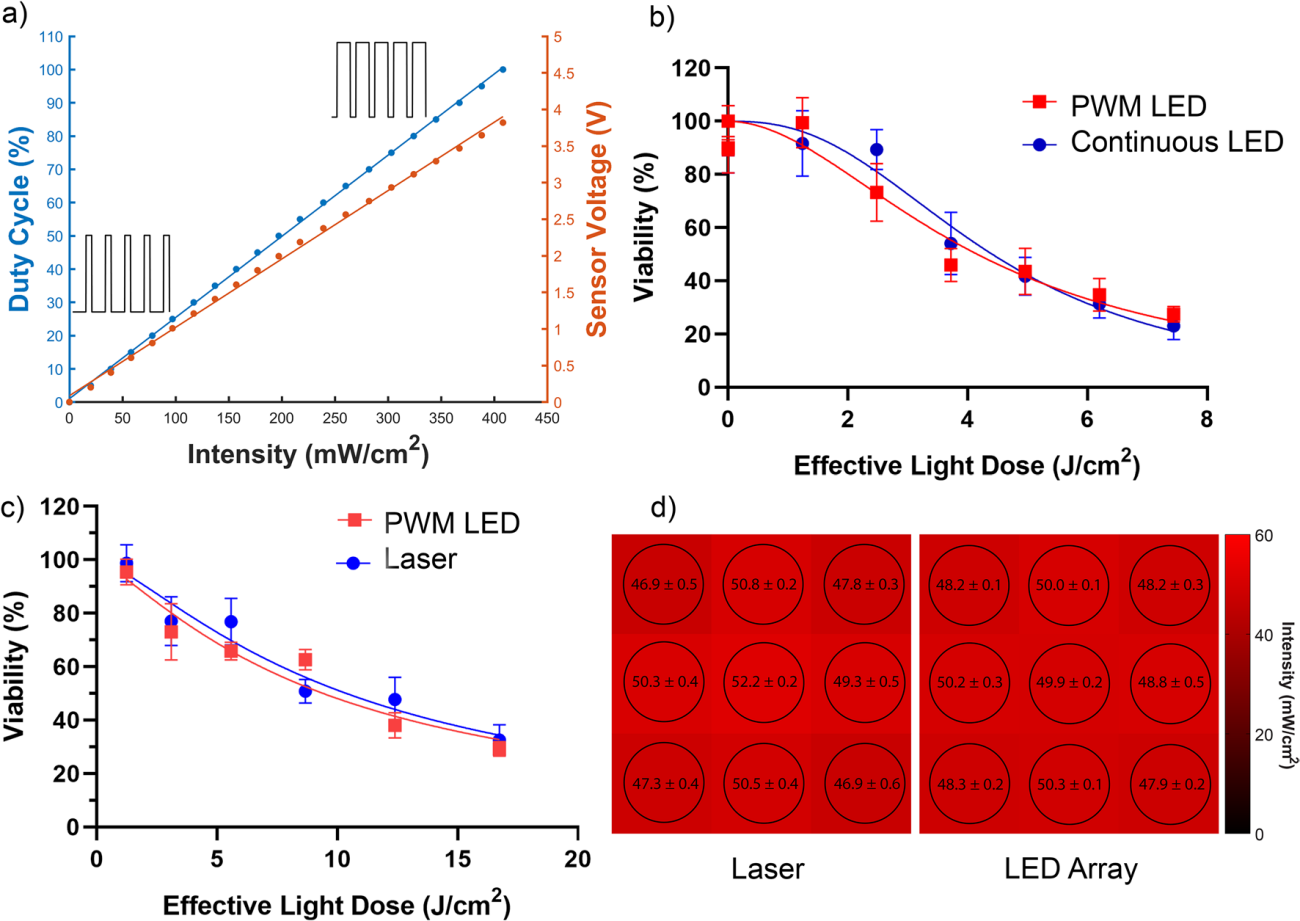
Validations of PWM-pulsed LED array operation for PDT. (**a**) The duty cycle of the PWM corresponds linearly to the power meter-measured intensity at sample plane as well as to the on-board phototransistor sensor circuit output voltage. Minimal deviations to linearity are due to saturation effects and these are of course calibrated for accurate control of the irradiance up to the maximum system output of 400 mW/cm^2^. (**b**) The light dose response comparison between the PWM-pulsed LED array and the voltage-adjusted continuous LED array for PDT of OVCAR3 cell cultures with the same mean irradiance (100 mW/cm^2^). The fit effective EC_50_ values are 4.16 J/cm^2^ (PWM) and 4.41 J/cm^2^ (continuous). **(c)** The light dose response comparison between the PWM-pulsed LED array and the laser. The fit effective light dose (calculation described in the text to account for the broadened spectrum of the LED array compared to the laser) EC_50_ values are 9.41 J/cm^2^ (PWM LED) and 10.38 J/cm^2^ (laser). (**d**) The laser (left) and LED (right) setup illumination power measured at the position of each well.

Here, we propose a simple constant voltage design using pulse-width modulation (PWM) to control the average power of the LEDs. In this design, high-current transistors are used in series with the LEDs as a fast switch. As the on–off time of the LEDs and the transistors are less than 100 ns^27,28^, the power output is proportional to the duty cycle, assuming the other parameters, such as environmental temperature, are the same. We have experimentally proven the linearity with an error of < 3 mW (Fig. 3a).

Though the light dose and the average power delivered to the sample are the same as a typical PDT setup, the peak power is increased substantially by the PWM method. Therefore, it is necessary to test the effectiveness of the PWM method. We measured the light dose–response of OVCAR3 cancer cells with verteporfin (VPF) as the photosensitizer. To compare, the LED array was also connected to a variable DC power supply, which was carefully adjusted to provide the same average power. The result shows a minimal difference between the pulsed light treatment and the continuous light treatment (Fig. 3b).

We further tested whether the difference in spectrum of the custom LED array versus a commercial diode laser (690 nm, 1.5 W, Modulight ML6500) results in a difference in light dosimetry. Prior reports have indicated that LED and laser dosimetry are roughly comparable when the LED spectrum overlaps strongly with the photosensitizer spectrum^29–31^. However, the increased spectral width of LEDs compared to lasers can result in a reduced spectral overlap integral with the photosensitizer absorption. Therefore, the LED irradiance is not equivalent to the same irradiance using a laser in terms of the actual PDT dose. Here, we measured the VPF absorption spectrum as well as the spectral response of the powermeter sensor, the LED array, and the 690 nm diode laser. Compared to the laser, the spectral efficiency of the LED array for VPF-PDT is calculated to be 61.98% (see Supplementary Material S2 for details regarding the calculation spectral overlap integral calculation and correction for the powermeter spectral response). To reflect the spectral efficiency of VPF absorption, and to make the PDT light dosimetry results comparable with laser-based PDT, the term Effective Light Dose can be defined to account for the LED spectral output overlap with the photosensitizer absorption spectrum relative to a laser line, which reflects the relative probability of photon absorption for PDT to estimate the photon deposition. With these considerations, the results indicate a similar effective EC_50_ of the laser-based and the LED-array-based PDT (10.38 vs. 9.41 J/cm^2^, Fig. 3c). To confirm the effectiveness, a fluorescence microscopic imaging with live/death stain is performed. Highly consistent live/death ratio and cell distribution is observed. The imaging results and methods are included in the Supplementary Material S3.

Another interesting observation is that the error bars of the LED-based PDT are generally smaller than laser-based PDT in the multiwell plate format. This effect is more apparent at where the slope of the dose response curve is steeper. This is likely due to the relatively large size of the LED array and wider emitting angle of the LEDs, resulting in a more uniform spatial light distribution compared to laser-based PDT, and hence produce less light dose variation over different wells. Further investigations have been done to measure the light intensity at the position of each well of a 3 × 3 matrix (the most common usage for experiments using a 96-well plate). The relative power difference between the center well and the least illuminated corner wells is 4.01% compared to the laser being 10.2% (Fig. 3d).

### Sample heat shielding and PCB water cooling stabilize thermal effects

**Figure 4:**
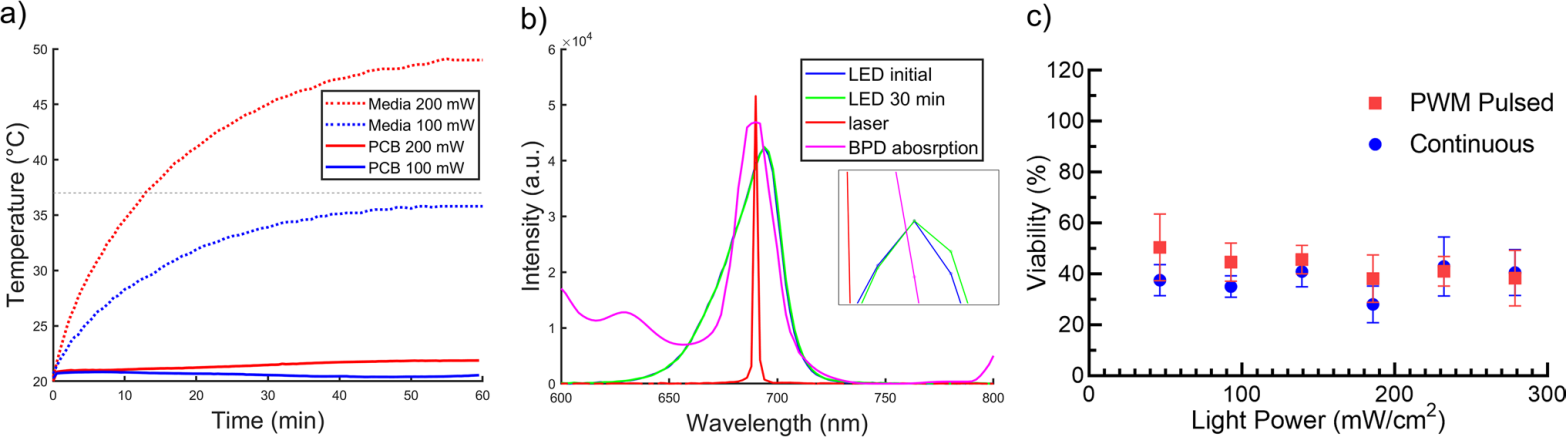
LED spectral stability and maximum irradiance in cell culture 96-well plates to avoid sample overheating and to obtain optimal PDT efficacy. (**a**) The measured temperature of cell culture media within the sample well plate (dotted) and the LED array PCB (solid) over time during the 60-min illumination test period, for a test run at 100 mW/cm^2^ (blue) and at 200 mW/cm^2^ (red). A line at the standard 37 °C incubation temperature (dashed grey) is included as a reference. (**b**) The output spectrum of the LED array before (blue) and after (green) a 60-min, 200 mW/cm^2^ illumination protocol. The spectrum is stable over the 1-h test period with only a sub-nanometer shift in peak wavelength visible in the zoomed inset. The spectrum of the 690 nm laser (red) and the BPD absorption spectrum (magenta) are also included for comparison. (**c**) Viability of the OVCAR3 cells after a*n* 8 J/cm^2^ PDT treatment with different irradiances.

We noticed that during high-power illuminations for PDT using the LED array, the large amount of energy deposited to the sample in a short time period could significantly increase the temperature of the sample. This occurs presumably through light absorption of the well plate material itself with some minimal contribution from water absorption within the cell culture media. This effect is apparently more significant in broader spectrum illumination, as the lower spectral overlap with the photosensitizer absorption requires a higher power, thus potentially higher thermal effect^32^. To help overcome sample overheating, a layer of aluminum foil is glued to the bottom of the spatial light filter that helps to reflect stray light away from the sample. We measured the temperature of the sample (i.e., the cell culture media in the wells) while continuously applying light until the temperature became stable (Fig. 4a). The test is performed in a temperature-controlled room, and the starting temperature is the room temperature 20.2 °C. At the typical^33–35^ PDT treatment illumination power of 100 mW/cm^2^, the temperature stops increasing at 35.8 °C, which is below but coincidently near standard cell culture incubation temperatures and actually desirable. At 200 mW/cm^2^, the sample temperature rises beyond 37 °C in 13 min, with a dose of 156 J/cm^2^, and saturates at 49.0 °C. Therefore, performing PDT at 100 mW/cm^2^ has no overheating effect, similar to laser PDT. However, higher powers cannot be used to expedite the experiment without overheating the cell culture to induce hyperthermic effects.

Another common concern is the temperature change of the LED, which leads to a shift in the light spectrum^25^. To solve this, a water-cooling loop is used to dissipate heat efficiently. The water-cooling loop consists of a water pump, a heat radiator, and the water block that is attached to the PCB by thermal paste. This concept is borrowed from the water cooler systems used broadly for high-performance computers. Water running in the loop carries the heat generated by the PCB to the radiator. Two 140 mm AC fans are used to further dissipate the heat from water to the environment. During the above 200 mW/cm^2^ test, we monitored the LED output spectrum. A total spectral shift of 0.2 nm is observed during the one-hour process (Fig. 4b). Compared with the spectral width of LED emission and the spectral width of the peak in BPD absorption near 690 nm (both are around 10 nm), the efficiency change due to this spectral shift is negligible.

### LED array enables high power and short irradiance times for in vitro PDT

The relatively high irradiance (400 mW/cm^2^) enabled by this new LED array design, compared to standard PDT irradiation setups^36^ (~ 10–150 mW/cm^2^) can potentially be used to shorten in vitro PDT experiments. However, higher irradiances could lead to the depletion of local oxygen, the efficacy of PDT could be affected and suboptimal especially in vivo since the oxygen concentration for in vivo tissues is lower than in in vitro cultures^37,38^. To test the efficacy in vitro, we performed a test that applies the same dose (8 J/cm^2^, near the EC_50_), but with varying irradiances up to 400 mW/cm^2^, to OVCAR3 cell cultures. The viabilities for all samples are between 38 and 50% (Fig. 4c), with no substantial trend with increasing irradiance (R^2^ < 0.2).

## Discussion

We introduce an easy-to-assemble PDT treatment illumination system for multiwell plate cell culture experiments with automated light exposures. The programmable robot arms automatically move and align the plate for each test group. In our experience for typical PDT experiments, the setup reduces the treatment duration by at least 30 min in comparison with our previous setup^25^ or a similar laser-based design, by removing the plate alignment and power adjustment processes. The operators are also released from waiting by the setup during the treatment. The automation process also removes the position and timing randomness, as well as mistakes, by human operation, so the treatment results are more precise.

The total cost of the setup (excluding the computer and the test instruments) is less than $1500. In comparison, a typical laser suitable for PDT is approximately $10,000. In addition, the modular design makes each part of the system replaceable, which significantly reduces the maintenance and modification cost. One possible scenario is to change the operating wavelength. Only a change of the LED array PCB is required (cost ~ $400). The modular design also offers high scalability to this setup. A larger PCB with more LEDs can be used for a larger and smoother illumination area. Another potential application is to use LEDs with different wavelengths on one PCB to perform multi-photosensitizer multiplexed PDT.

To the best of our knowledge, the PDT results from the incoherent sources are seldomly compared with the laser-based PDT, due to the different spectral efficiencies. With the correction of the photosensitizer absorption and the powermeter sensitivity spectra, the LED-based PDT results are highly consistent with the laser. This enables the laboratories using LEDs to compare their results with the more available laser results in the literature.

That said, this setup is not a mature, commercial product, and many aspects can be improved. The robot arms are made of 3D-printing PLA, which is not rigid enough if a heavier well-plate is used. Machined metal arms can be a potential improvement. Also, the lack of position feedback means the arms will not correct automatically should a position error happen, such as an accidental touch when the motors are not powered. In these cases, a manual position reset is required. In addition, the lack of a temperature control system on the sample well plate means the light irradiance is limited to around 100 mW/cm^2^ on experiments involving temperature-sensitive cell lines and drugs. The power difference between the wells is up to 4%, which is significantly better than our attempt to flatten the gaussian beam profile of the laser. That said, there is still room to improve the uniformity of LED sources and it may be possible to reach significantly more uniform illumination with future design tweaks.

The setup is designed for a quick and easy way to apply light treatment to cells in the well-plates. It is most suitable to test photomedicine in the early in vitro developing stages. While the laser is so far the best choice for precise, in vivo, and fiber-coupled operations, the lower cost, capability to switch wavelengths, high throughput, and the ease to fabricate this setup may be attractive to some PDT researchers.

## Methods

### LED array PCB design

The LEDs used are high power and 5-by-5 mm, featuring a lens that converges most of the light within 24 degrees (SMBB690D-1100-03, Ushio). Each LED is supplied with 2.4 V DC when powered on, with forward current of 600 mA and output power of 520 mW^27^. The overall size of the array is 33 × 33 mm.

The total current drawn from the LED array is about 3 A. To reduce the power loss and heat effect due to the resistance of the PCB leads, a large cross-section is required. With 4 mm lead width and 0.07 mm PCB copper layer height. The cross-section of the lead is equivalent to that of an AWG 23 wire. The voltage difference across the power lead is less than 0.04 V at 3 A, which is around 0.3% of the voltage across the penta-LED series. The power of the LED is provided by a computer power supply with the 12 V ATX output. This minimizes the Ohmic loss of the circuit and, more importantly, provides the same voltage to each LED to guarantee a uniform light distribution.

Two metal–oxide–semiconductor field-effect transistors (MOSFETs) are used as the switch to control the current. The two MOSFETs are connected in parallel to share the current burden. The nanosecond-level switch time of the MOSFETs is essential for PWM switching. Their relatively small profile enables them to be integrated into the PCB, making them the ideal choice over mechanical relays, despite the latter having a typically larger current rating.

The sensors circuit uses the fixed-value resistors (5 kΩ for thermistor and 10 kΩ for phototransistor) to form voltage-divider circuits. The values are chosen to create output voltage ranges with magnitudes around multiple volts. This maximizes the resolution and avoids overshooting the 10 V measuring range of the DAQ digitizer.

On the PCB, the control lead connecting the gate of the MOSFETs is placed through the gap between the source and drain terminals. In this way, the entire circuit can be printed in one layer. This enables using aluminum as the backbone of the PCB, which conducts the heat to the waterblock effectively.

### Wiring the electronics and water-cooling loop

The MOSFETs’ base pin is connected to the analog output port (AO0) of the DAQ controlled by the computer. The DAQ generates a 250 Hz square wave, with a 10V_pp_ amplitude and 100% offset, which is equivalent to a 0–10 V pulse. With the 5 kHz sampling frequency, each wave period consists of 20 sample cycles. Therefore, the duty cycle of the square wave can change in steps of 5%, providing 20 different irradiance levels.

The sensors are connected in series with their corresponding resistors (5kΩ for thermistor, and 10kΩ for phototransistor). The outputs are connected to the analog input ports (AI0 for thermistor and AI1 for phototransistor). With the PWM control mentioned above and the common ground, the four wires form cable 1 connecting the PCB and the DAQ.

**Figure 5:**
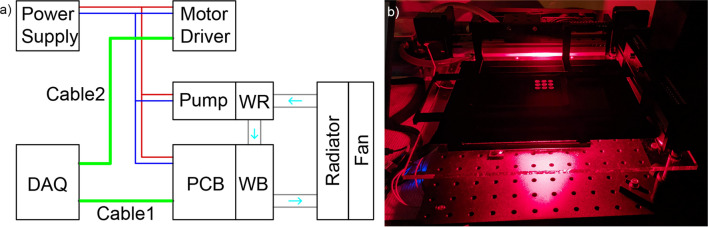
(**a**) The electrical wiring diagram of the system. DAQ, data acquisition board; PCB, LED array printed circuit board; WB, water block; WR, water reservoir. Cable1: 4-wire analog cable consisting of PWM control, thermistor signal, ground, and phototransistor signal. Cable2: 3-wire digital cable consisting of stepper motor pulse control, direction control, and ground. Note the other motor driver and its control cable (same as Cable2) is not shown to simplify the diagram. The cyan arrows indicate the water flow direction. (**b**) The LED array system in operation during a 96-well plate PDT experiment. Note the opaque cover above the plate is removed to show the illuminated 3 × 3 wells in this demonstration.

Most of the components (the LED array, the sensors, the robot arm stepper motor drivers, and the water pump) use 12 V DC provided by the ATX power supply. The ribbon cable consists of AWG 14 wires, which conducts large current with minimal heat effect. The LED array PCB is connected to the dual + 12 V output (conventionally used to power the CPUs) with the Molex connecter which brings ease when switching the board. The sensors are powered by the same 12 V power as the LEDs. This is achieved by the PCB circuit connection. The water-cooling pump and the stepper motor drivers are powered using the 12 V and GND pins of the peripheral output with custom AWG 22 wires. An illustration of the wiring is shown in Fig. 5.

The stepper motors of the robot arms are connected to their corresponding driver modules with a proprietary 4-wire cable, through which both driving signal and power are transmitted. The driver module is controlled by the DAQ via low-voltage (5 V) digital signals. A 4-wire cable is used to connect the PUL + (to p0.1 on DAQ) for motion-stop control pulses, the DIR + (to p0.2 on DAQ) to control the direction of the motion, and the grounds (PUL-, DIR-, to GND on DAQ). The ENA + /− ports are directly connected to the 12 V power and the corresponding ground ports, as the motors are always enabled. The stepper motor-driver module for the other direction is connected in the same way (using the DAQ p0.3 and p0.4 ports respectively).

### Optics assembly with 3D-printed mounts

**Figure 6:**
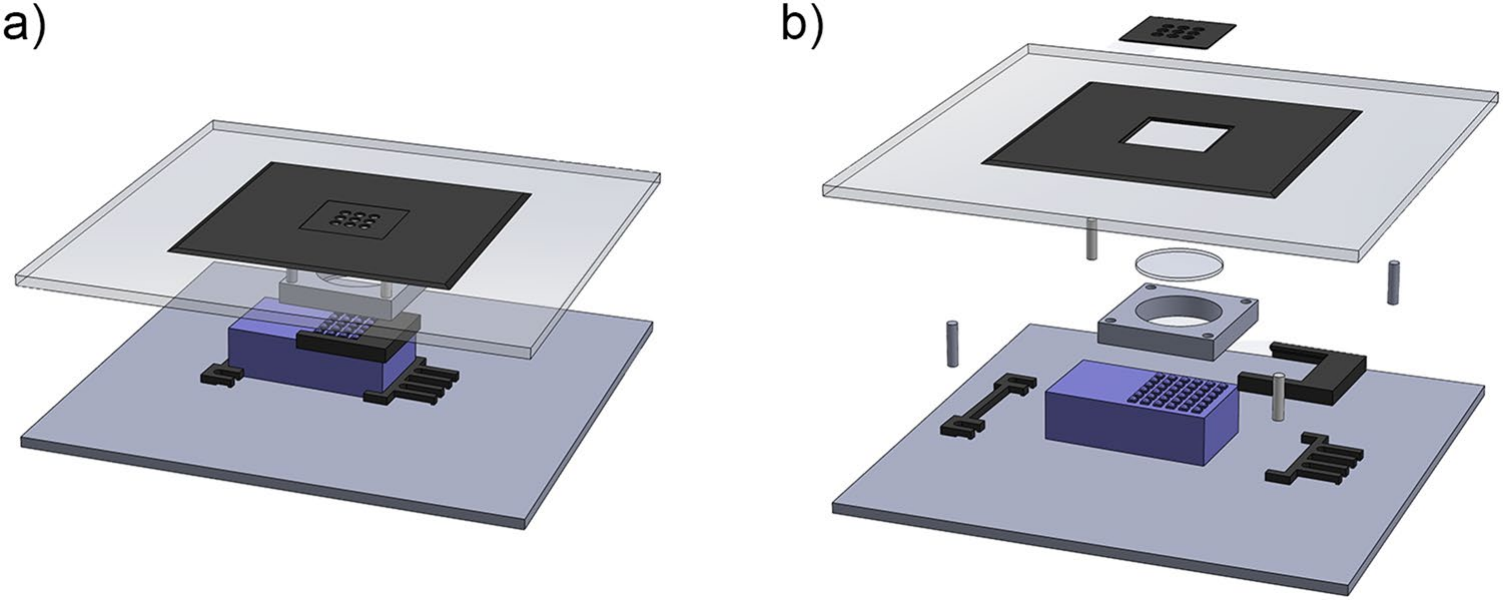
(**a**) The 3D model of the optical assembly. The 3D printed parts are colored dark grey. The LED array module is colored blue. The bottom plate is the optical breadboard. The top plate is the acrylic platform. The black masking tape covering the platform, the robot arms parts, and the optomechanical mount of the platform are not shown. (**b**) The exploded view of the assembly to show the individual parts.

The combo of the LED array and the water block forms the source part of the optics. A 3D printed mount is used to hold the PCB and the water blocks and provide a flat bottom to mount the module onto the optical breadboard. Four adjustable rubber feet are placed under each corner of the breadboard to keep it level and isolated from the vibration caused by the water cooling system. Two more pieces of 3D-printed bracket are used to fix the position on the optical breadboard with ¼ inch screws. Another 3D-printed clip is used to align the PCB to the center of the water block to keep the optical alignment (Fig. 6). Therefore, the PCB can be easily changed if a change in wavelength is required.

The 2-inch Fresnel lens is held 1 inch above the LEDs by four cage system rods. The distance 1 inch is determined experimentally to maximize the light intensity at the sample plane while not compromising the uniformity. The rods are attached to the bottom of the platform through the through-hole flat screws.

### Cell cultures, performing PDT and viability measurement

Human epithelial ovarian cancer cell line NIH:OVCAR-3 (OVCAR3) was purchased from American Type Culture Collection (ATCC, HTB-161™) and cultured in T75 flasks (Fisherbrand™, FB012937) in a humidified incubator at 5% CO2 and 37 °C. OVCAR3 cells were maintained in RPMI 1640 Medium (Gibco™, 61-870-127) with 20% heat-inactivated FBS (R&D Systems, S11150H), 1% penicillin/streptomycin (Fisher BioReagents, BP295950) and 0.01 mg/ml bovine insulin (Sigma Aldrich, I0516). Cells were passaged at 80–90% confluency; TrypLE™ Express Enzyme (ThermoFisher Scientific, 12,604,021) was used for lifting the cells. Cells between the 5th and 30th passages were used for the experiments.

Before plating cells for PDT experiments, cells were suspended at 20,000 cells/ml in the complete growth medium. 100 μL of cell suspension was added to 90 wells of a black-walled flat-bottom 96-well plate (PerkinElmer, 6,055,300) and 100 μL complete growth medium with no cells was added to the remaining 6 wells as the Media Only control. On day 4, old media was removed by inverting the plate. Then the drug is added to different treatment groups with media as shown in Fig. 2b. Each plate had 16 groups, including 12 treatment groups, three control groups (drug with no light (DNL), light with no drug (LND), and no light with no drug (NDNL), and one media only (MO) group where no cells present) and all the groups had six replicates. The photosensitizer is added with 100 μL fresh media to all 12 treatment groups and DNL group, while the fresh media without the photosensitizer is added to the LND, NDNL, and MO groups. The well plate is then incubated for 1.5 h before applying light illumination.

In the effectiveness comparison experiments (PWM LED vs continuous LED, and PWM LED vs laser), the PWM controlled, LED-based therapy is performed by the setup mentioned above. The continuous LED therapy is performed by replacing the computer power supply with a variable DC supply and keeping the PWM duty cycle being 1. The laser therapy is performed by replacing the LED array with a fiber-coupled diode laser (ML6500, Modulight). The output of the fiber is placed on the focal point of the Fresnel Lens. Due to the power limitation, the PWM LED vs laser experiment is performed at 50 mW/cm^2^. The dose applied to each series are 2, 4, 6, 8, 10, and 12 J/cm^2^. The LND control is treated with the PWM controlled LED with a dose equal to the maximum dose applied to a treatment group (12 J/cm^2^) and power equal to the treatment groups (100 mW/cm^2^ for PWM vs continuous, 50 mW/cm^2^ for LED vs laser). A video demonstration of a routine use of this setup is available at https://youtu.be/t2Nl7pCarnA.

In the effect of power experiments, the optics setup is the same as the PWM vs continuous test mentioned above, except the robot arm is not being used. The light power applied to each series is 45, 90, 135, 180, 225, 270 mW/cm^2^, and the light dose is 8 J/cm^2^ for all groups. The light power is measured by the power meter before performing treatment on each group. The LND control group is treated by light dose 8 J/cm^2^ at irradiance 270 mW/cm^2^.

The well plate is incubated for 72 h after the illumination. Then the cell culture viability was measured using CellTiter-Glo® Luminescent Cell Viability Assay (Promega, G7570). The plate and its contents were equilibrated at room temperature for 30 min. CellTiter-Glo reagent was formulated by mixing CellTiter-Glo® Buffer and CellTiter-Glo® Substrate equilibrated at room temperature. 100 μL reagent was added to each well and mixed the contents on an orbital shaker (DragonLab, SK-O180-E) for 2 min. The plate was then incubated at room temperature for 10 min and the unfiltered luminescence was recorded by using a plate reader (BioTek, Synergy LX Multi-Mode Reader) at 1 s integration time. Plate reader data was further analyzed by using GraphPad Prism. NDNL control group was used to define 100% viability and MO group was used to define 0% viability. EC50 values were determined using a nonlinear fit (Inhibitor vs. Normalized response—Variable slope) on GraphPad Prism.

## Supplementary Information

Supplementary Information 1.

Supplementary Information 2.

Supplementary Information 3.

Supplementary Information 4.

Supplementary Information 5.

Supplementary Information 6.

Supplementary Information 7.

Supplementary Information 8.

Supplementary Information 9.

## Data Availability

The source code of the control software used is available at https://github.com/springlabnu/pdtV4.
