# Supplementary material for: An open-source LED array illumination system for automated multiwell plate cell culture photodynamic therapy experiments
Source: Sci Rep. 2022 Nov 11;12:19341. doi: 10.1038/s41598-022-22020-7 (PMC9652332; doi:10.1038/s41598-022-22020-7)
Supplement: Supplementary file 8 — Supplementary Information 8. [file 41598_2022_22020_MOESM8_ESM.docx]

# Supplementary Materials: Automated and stable LED array illumination system for multiwell plate cell culture photodynamic therapy experiments

Kai Zhang, Sudip Timilsina, Matthew Waguespack, Eric M. Kercher, and Bryan Q. Spring

## S1. Well plate layout

Fig. 2b shows a typical layout to measure the light-dose response for two different treatment groups. The cells of the groups are in the same plate during the culturing, drug application, and viability measurement processes to minimize the error due to the environmental difference.


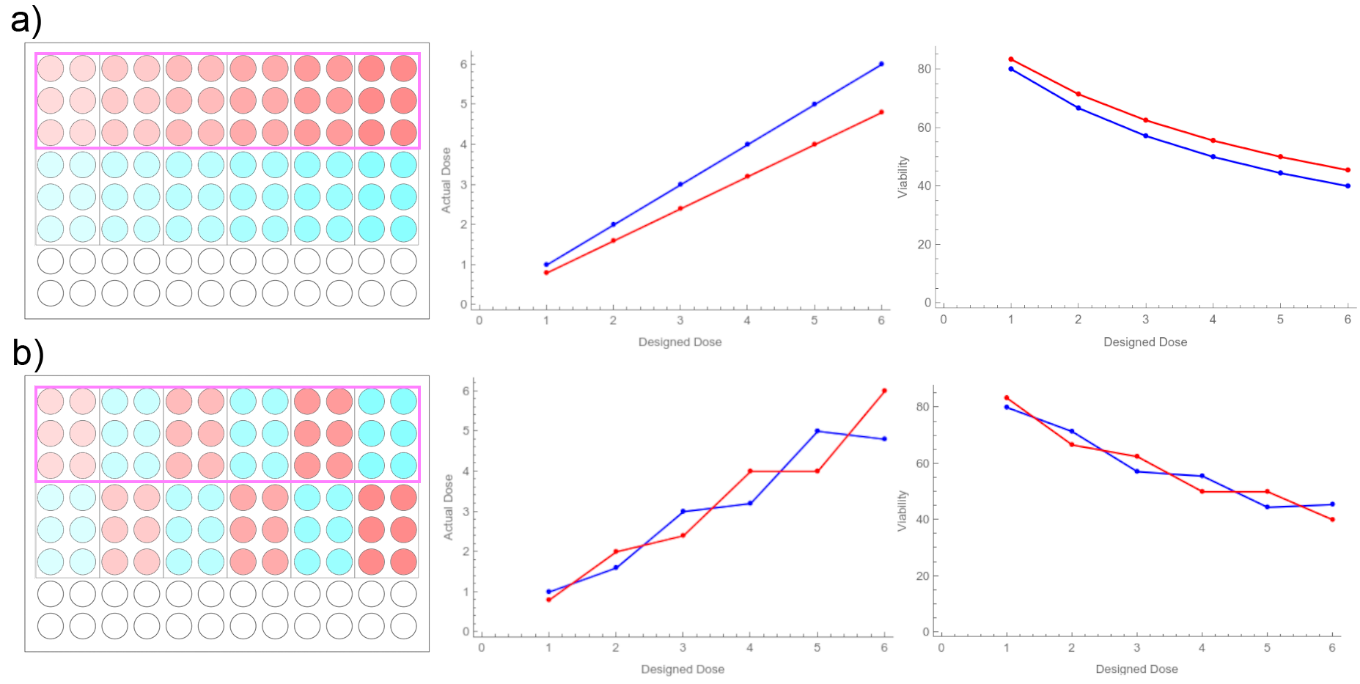


**Fig. S1** The chessboard pattern is used to detect the misalignment caused by the robot arms. **(a)** In a rare case that the wrong calibration profile was used, the misalignment could happen to a row of treatment group with the same amount. Therefore, wells in this misaligned row (assume the wells highlighted by magenta rectangles) would receive a proportionally different light dose than the experimental design (middle). This could be misinterpreted as a different EC_50_ in the light dose response curve. If the checker pattern was used as shown in **(b)**, light dose applied to the groups in the same set would change differently, resulting an easy-to-detect saw-tooth-like pattern in the light-dose response curve, indicating a bad alignment. The data in the plots here are for demonstration purposes only.

**S2. Derivation of LED spectral efficiency**

Define $I_{L} \mathrm{and} I_{D}$ as the actual intensity of the laser and the LED, respectively. Define $S_{L}\left( \lambda\right)\mathrm{and}S_{D}\left( \lambda\right)$ as the spectrum of the laser and the LED, respectively; *i.e.*, $\int S{}_{i\left( \lambda\right)}d\lambda=I_{i}$, where the subscript *i* is *L* for laser and *D* for LED.

Define $A_{P}\left( \lambda\right) \mathrm{and}A_{S}\left( \lambda\right)$ as the absorption spectrum (extinction coefficient spectrum) of the power meter and the photosensitizer, respectively. The overlap integral of the light source output and the photosensitizer absorption gives the power absorption of the photosensitizer, $R_{S,i}=I_{i}\int S_{i}(\lambda)\times A_{S}(\lambda) d\lambda$, while the rest of the photons are transmitted and eventually become heat. The reading of the power meter $R_{P,i}=I_{i}\int S_{i}\left( \lambda\right)\times A_{P}\left( \lambda\right) d\lambda$

The ratio of the powermeter reading of the LED and the laser can be expressed by the actual power ratio:

$$\frac{R_{P,D}}{R_{P,L}}=\frac{I_{D}\int S_{D}\left( \lambda\right)\times A_{P}\left( \lambda\right)d\lambda}{I_{L}\int S_{L}\left( \lambda\right)\times A_{P}\left( \lambda\right)d\lambda}$$

Therefore,

$$\frac{I_{D}}{I_{L}}=\frac{R_{P,D}}{R_{P,L}}\frac{\int S_{L}\left( \lambda\right)\times A_{P}\left( \lambda\right)d\lambda}{\int S_{D}\left( \lambda\right)\times A_{P}\left( \lambda\right)d\lambda}$$

The ratio of the photosensitizer photon absorption between the LED and the laser is:

$$\frac{R_{S,D}}{R_{S,L}}=\frac{I_{D}\int S_{D}\left( \lambda\right)\times A_{S}\left( \lambda\right)d\lambda}{I_{L}\int S_{L}\left( \lambda\right)\times A_{S}\left( \lambda\right)d\lambda}$$

$$=\frac{R_{P,D}}{R_{P,L}} \frac{\int S_{L}\left( \lambda\right)\times A_{P}\left( \lambda\right)d\lambda}{\int S_{D}\left( \lambda\right)\times A_{P}\left( \lambda\right)d\lambda} \frac{\int S_{D}\left( \lambda\right)\times A_{S}\left( \lambda\right)d\lambda}{\int S_{L}\left( \lambda\right)\times A_{S}\left( \lambda\right)d\lambda}$$

While the LED and the laser spectra can be measured by a spectrometer, the power meter (S132C, Thorlabs) response and the absorption spectrum of VPF are available^39,40^. The numerical value of the four integrals can be calculated by programming with a discrete Reiman Sum technique. Note that absolute values and normalizations of the spectra are not required as these factors cancel out of the relative ratio.


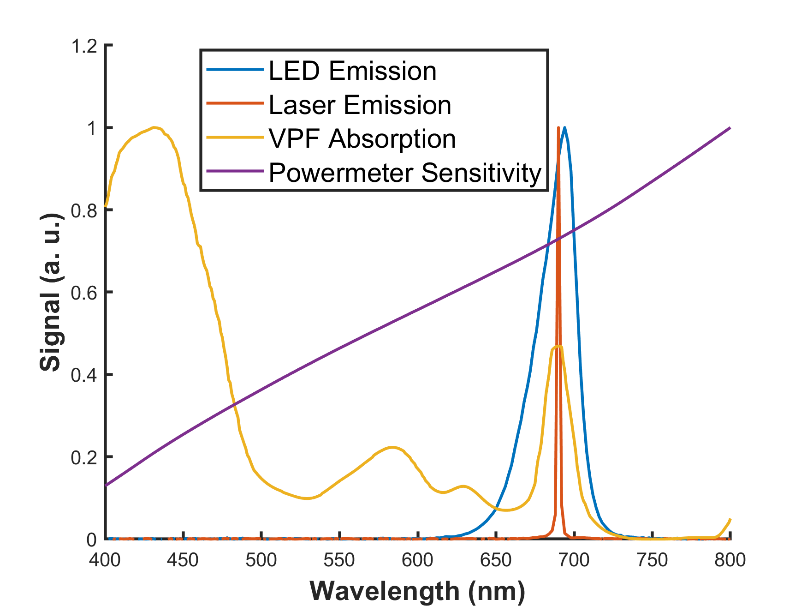


**Fig. S2** The normalized spectra of the LED (blue) and the laser (orange), the absorption spectrum of VPF (yellow), and the spectral response of the powermeter sensor (purple).

**S3. Fluorescence live/dead imaging procedures and results**

OVCAR3 cells and pre-PDT treatments are used as in the **Cell Cultures** methods part. The plate includes 8 groups, including 4 treatment groups (8 and 12 mJ/cm^2^, for both LEDs and lasers) and 4 control groups (DNL, NDNL, laser LND, LED LND). PDT illumination is performed with the same procedures as the effectiveness comparison experiments described in *Cell Cultures, Performing PDT, and Viability Measurement* methods part.

The well plate is incubated for 24 hours after the illumination. The wells were then washed with DPBS (Gibco™, 14190144) after discarding the supernatant. 1:60 dilution of acridine orange (AO)/propidium iodide (PI) (Logos Biosystems, F23001) was prepared in media and 50 µL of diluted AO/PI stain was added to each well and the cells were incubated for 20 minutes at 37°C before imaging.

Then the fluorescence microscopic imaging is performed with the confocal microscope (LSM 800 with Airyscan, Zeiss). The focal planes are chosen to be at the bottom of each well. The fluorescent images of the regions with similar relative position in each well are taken. The imaging results are adjusted with the same curve (software: Zen Blue, Zeiss) and crop region (software: Matlab, MathWorks).


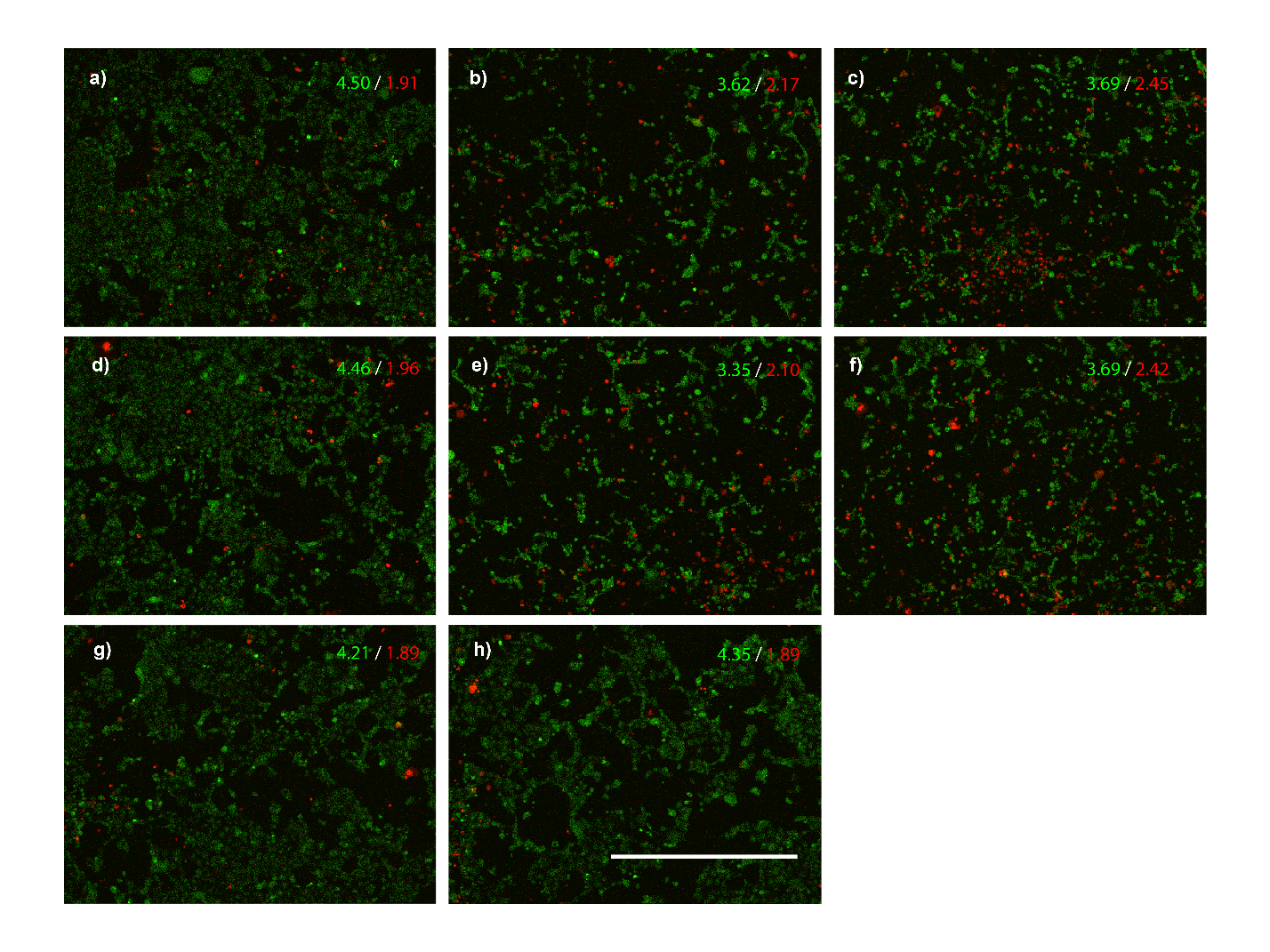


**Fig. S3** The fluorescence live/dead imaging results from LED-based PDT (top row) and laser-based PDT (middle row) with their histograms. (a) 12 mJ/cm^2^ LED light no drug control, (b) 8 mJ/cm^2^ LED illumination PDT, (c) 12 mJ/cm^2^ LED illumination PDT, (d-f) the corresponding laser parts, (g) DNL control, (h) NDNL control. Green, live fluorescence signal; red, dead fluorescence signal. The green/red numbers are the corresponding integrated live/dead signals (arbitrary unit). All images have same microscope settings, curve, and scale. Scale bar, 1mm.

**S4. Studies about the spectral shift due to the change in the LED temperature**

It is necessary to keep the LED temperature constant to stabilize the emission spectrum and power. A test is performed with the cooling water pump being turned off and the PCB temperature being raised up to 50 °C. A total peak wavelength shift of 4 nm is observed. The spectral efficiency is consequently reduced to 55.41% as calculated by the method in **S2**. The normalized spectra for different temperatures are shown in Fig. S4.


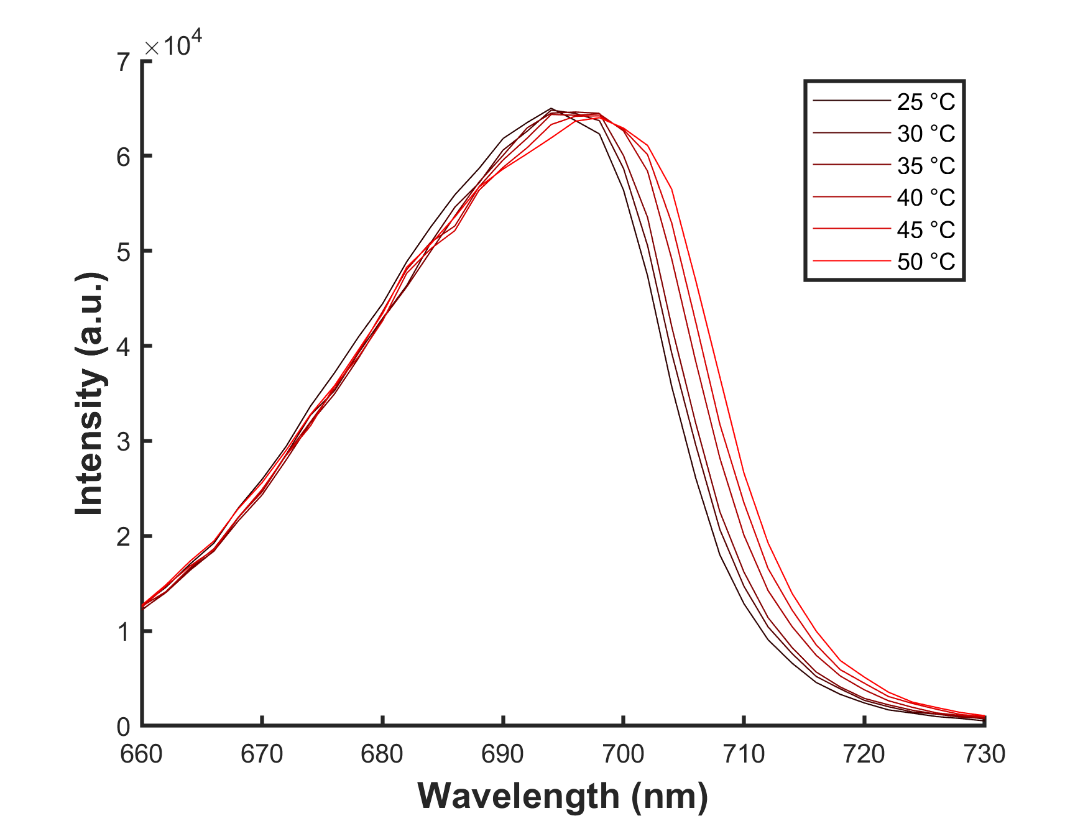


**Fig. S4** The 690 nm LED emission spectra at different temperatures.

**List of materials**

**Table S1.** The list of materials used to assemble the PDT setup

| **Item** | **Company** | **Part No.** | **Notes** |
| --- | --- | --- | --- |
| **Electronics** | | | |
| LED | Ushio | SMBB690D-1100-03 | 690 nm |
| MOSFET | Infineon | IAUC120N04S6L005ATMA1 |  |
| Thermistor | TDK Electronics | B57452V5103J062 |  |
| Phototransistor | Rohm Semiconductor | RPM-075PTT86 |  |
| Resistors | Stakcople Electronics | RNCP0805FTD****CT-ND | 4K99, 20K0, 10K0 |
| Connector Header | Sullins Connector | GBC06SGSN-M89 |  |
| Printed Circuit Board | PCB Way |  | Custom circuit, Aluminum |
| **Optical and Optomechanical Parts** | | | |
| Optical Breadboard | Thorlabs | MB12 | 12" × 12" × 1/2" |
| General Optomechanics | Thorlabs | 3× UPH2, 3× TR3, ER2-P4 |  |
| Cage Plate | Thorlabs | LCP01 |  |
| Fresnel Lens | Thorlabs | FRP232 |  |
| Diffuser | Thorlabs | DG20-600 |  |
| Black Masking Tape | Thorlabs | T205-1.0 |  |
| 3D-printing filament | Hatchbox |  | Polylactic Acid, Black |
| **Cooling and Robot Arm Parts** | | | |
| Acrylic Board | SimbaLux |  | 12" × 12" × 0.24" |
| Stepper Motor Actuator | RATTMMOTOR | CBX1605-200A | 2× |
| Stepper Motor Driver | STEPPERONLINE | DM860T | 2× |
| Multifunction I/O Device | National Instruments | USB-6001 |  |
| Water Cooling Pump | Bewinner | PUB-ST1000 |  |
| Water Block | Yibuy | YBY20180911 | 2× |
| Radiator Fan | AC Infinity | Axial 1238 |  |
| Radiator | Thermaltake | Pacific R240 | 240 mm |
| ATX power supply | Cooler Master | MPE-7501-AFAAG |  |
| ATX CPU power cable | COMeap |  | 9.5", male to female |
| **Test Instruments** | | | |
| Powermeter | Thorlabs | S130C |  |
| Powermeter Console | Thorlabs | P100 |  |
| Thermometer | Gain Express | 68022 |  |
| Laser | Modulight | ML6500 | 690 nm, 1.5 W |

## Pictures of the optical mounting components

##
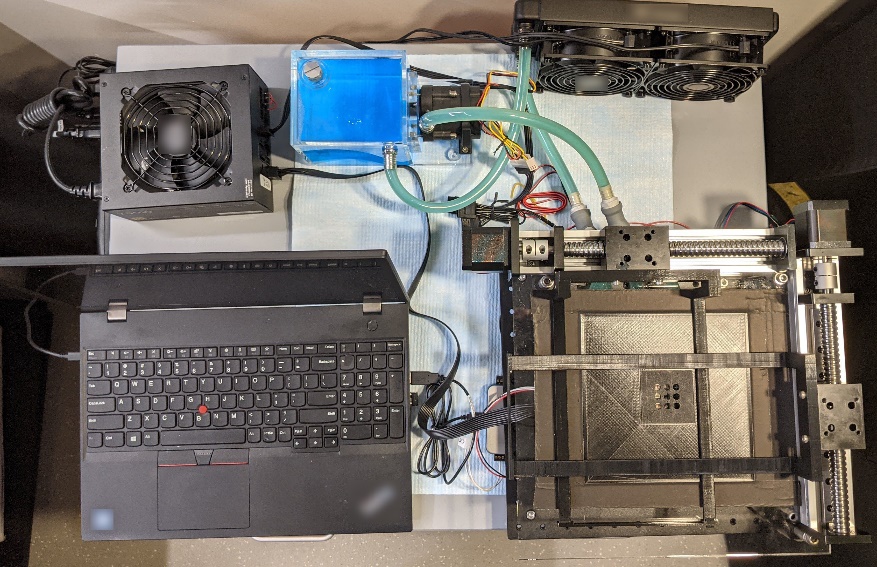


**Fig. S5** Top view of the full setup.

##
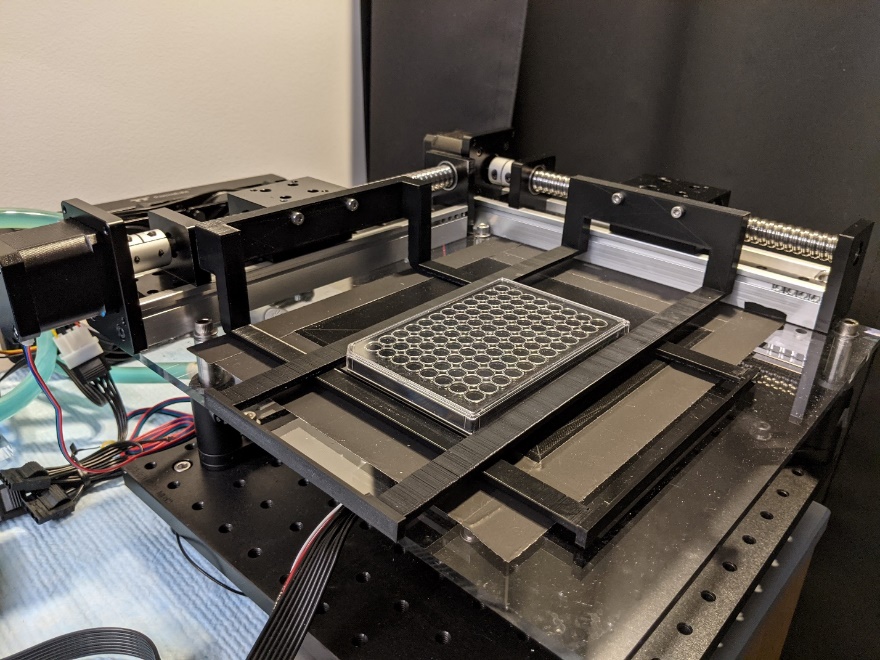


**Fig. S6** The PDT platform, with a 96-well plate loaded.

##
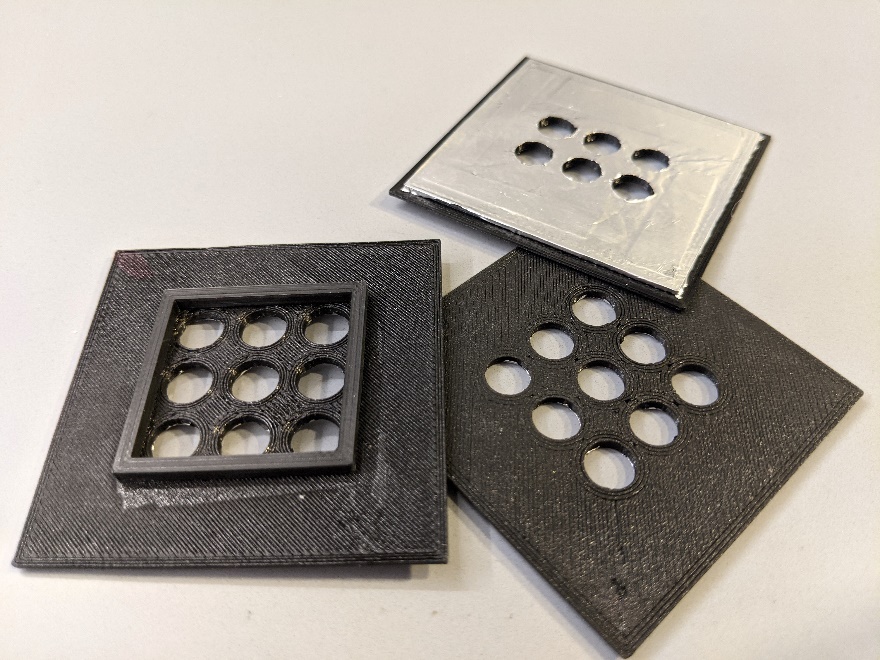


**Fig. S7** The spatial light filters for a 3×3 group in a ultra low attachement (ULA) plate (left), a 3×3 group in a flatt-bottom plate (lower right), and a 2×3 group in a flat bottom plate (upper right). Notice the upper right filter is flipped bottom up to show the aluminum heat shield.

##
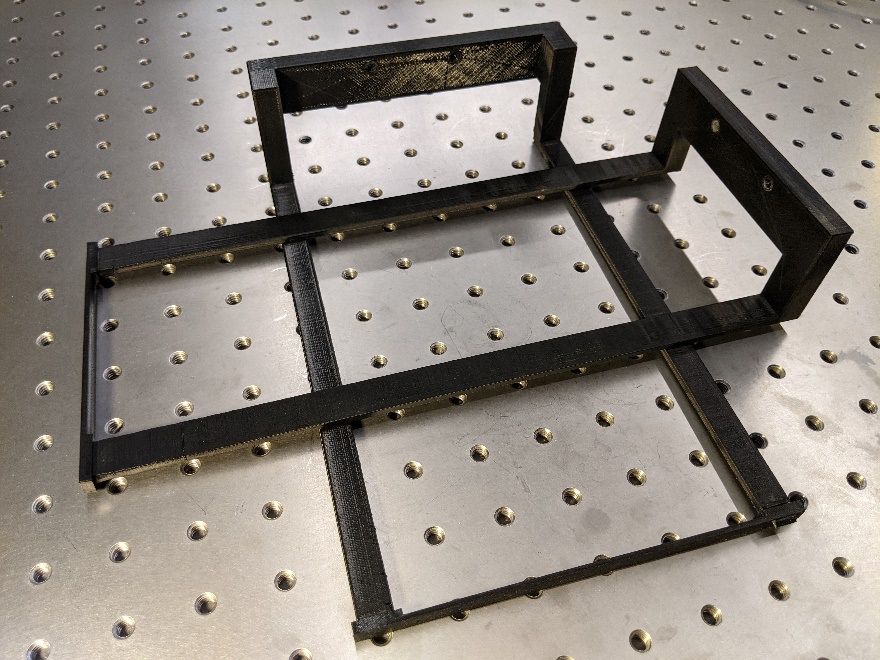


**Fig. S8** The two 3D-printed robot arms.

##
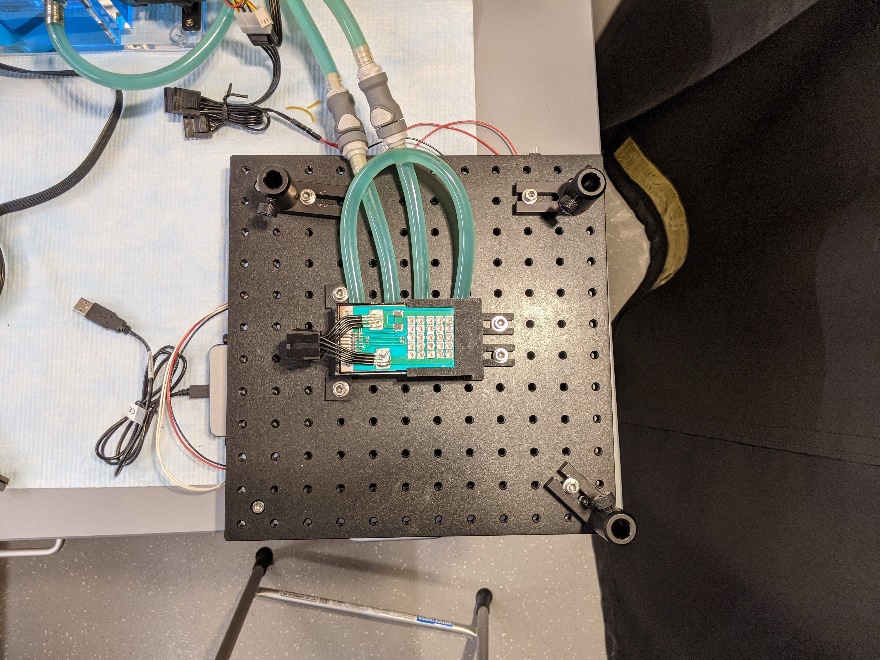


## Fig. S9 The LED array module under the well plate platform

##
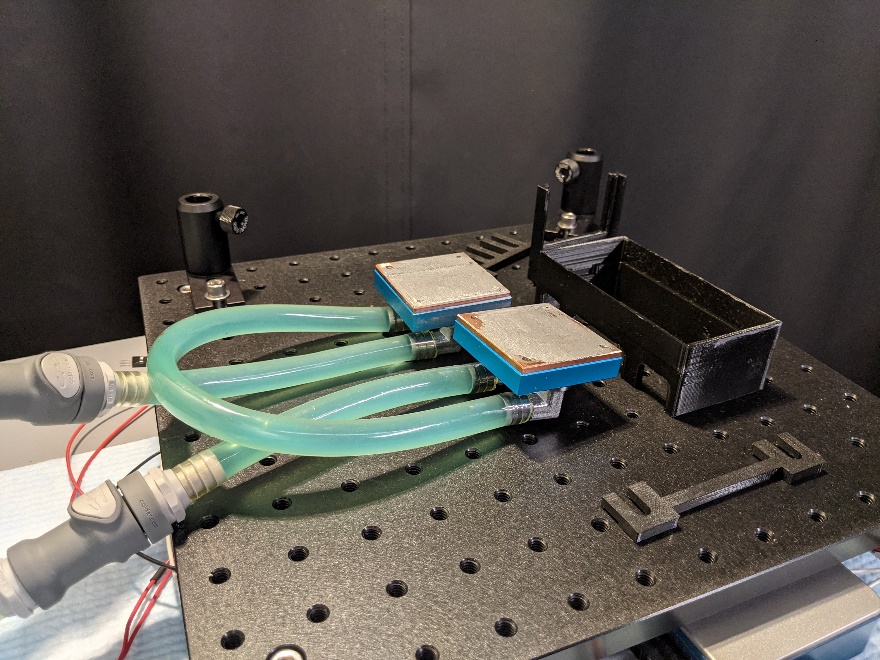


## Fig. S10 The individual parts of the LED array module.

##
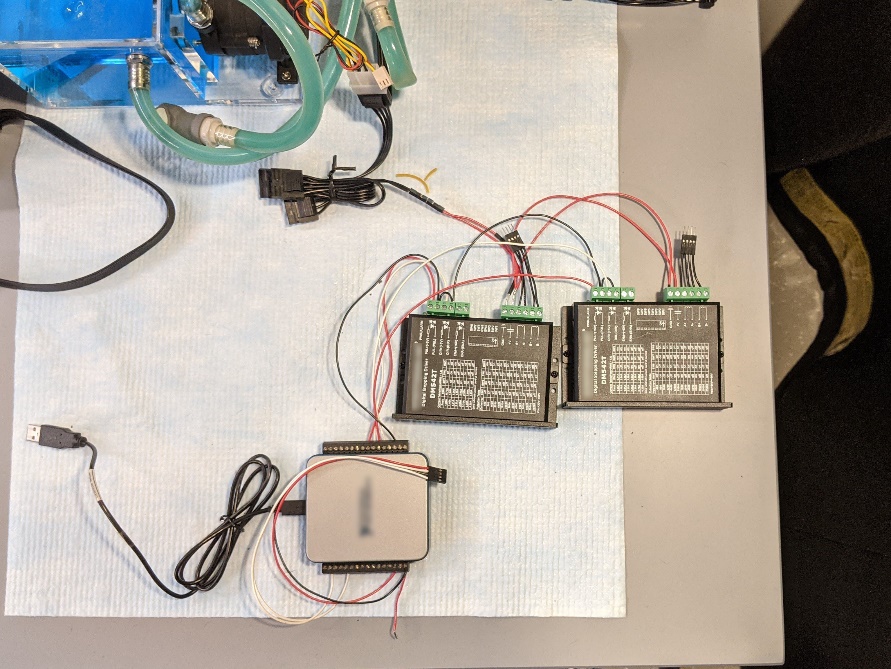


## Fig. S11 The controller (grey) and the stepper motor driver modules (black) under the optical breadboard

##
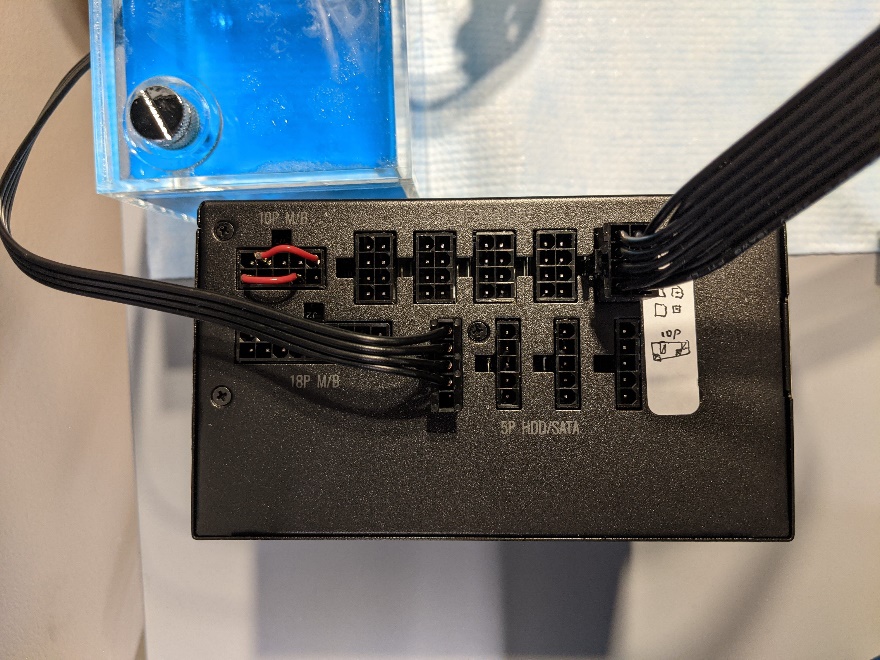


## Fig. S12 The wiring of the ATX power supply. The two jumper wires (red) are used to keep the power on without a computer motherboard.

**Supplementary Video Legend**


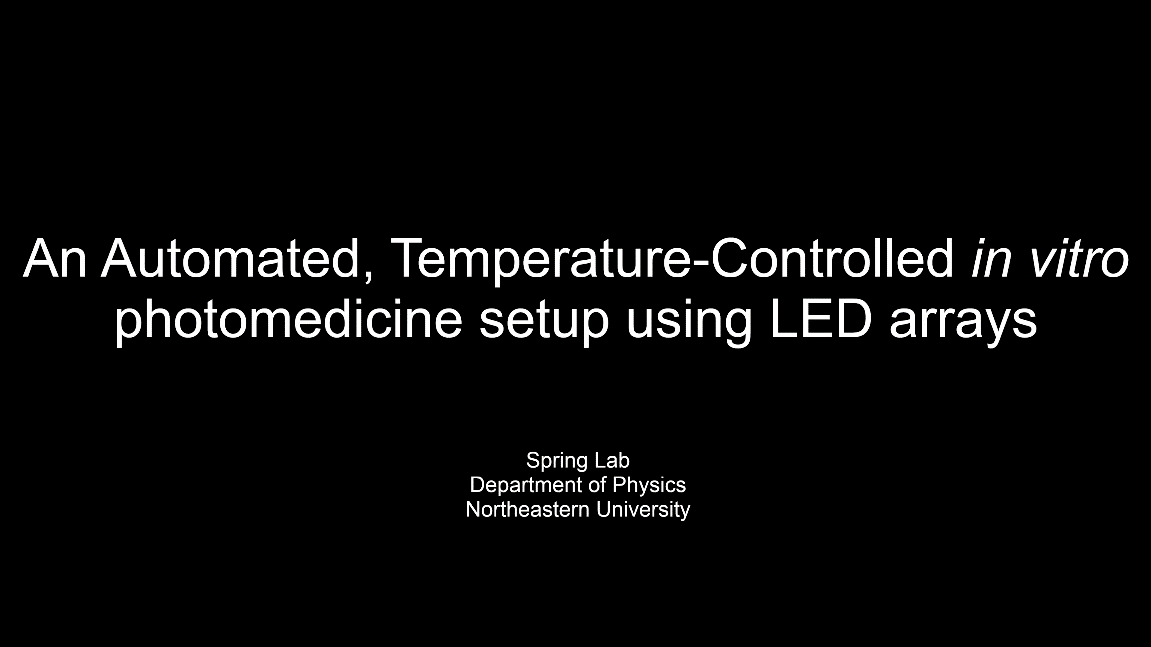


**The demonstration of the operation of the LED array illumination system.** The video shows the routine use of the illumination system with a 96-well rounded-bottom ultra-low-attachment cell culture plate as well as the regular robot arm position calibration.

**Supplementary Code Legend**

**
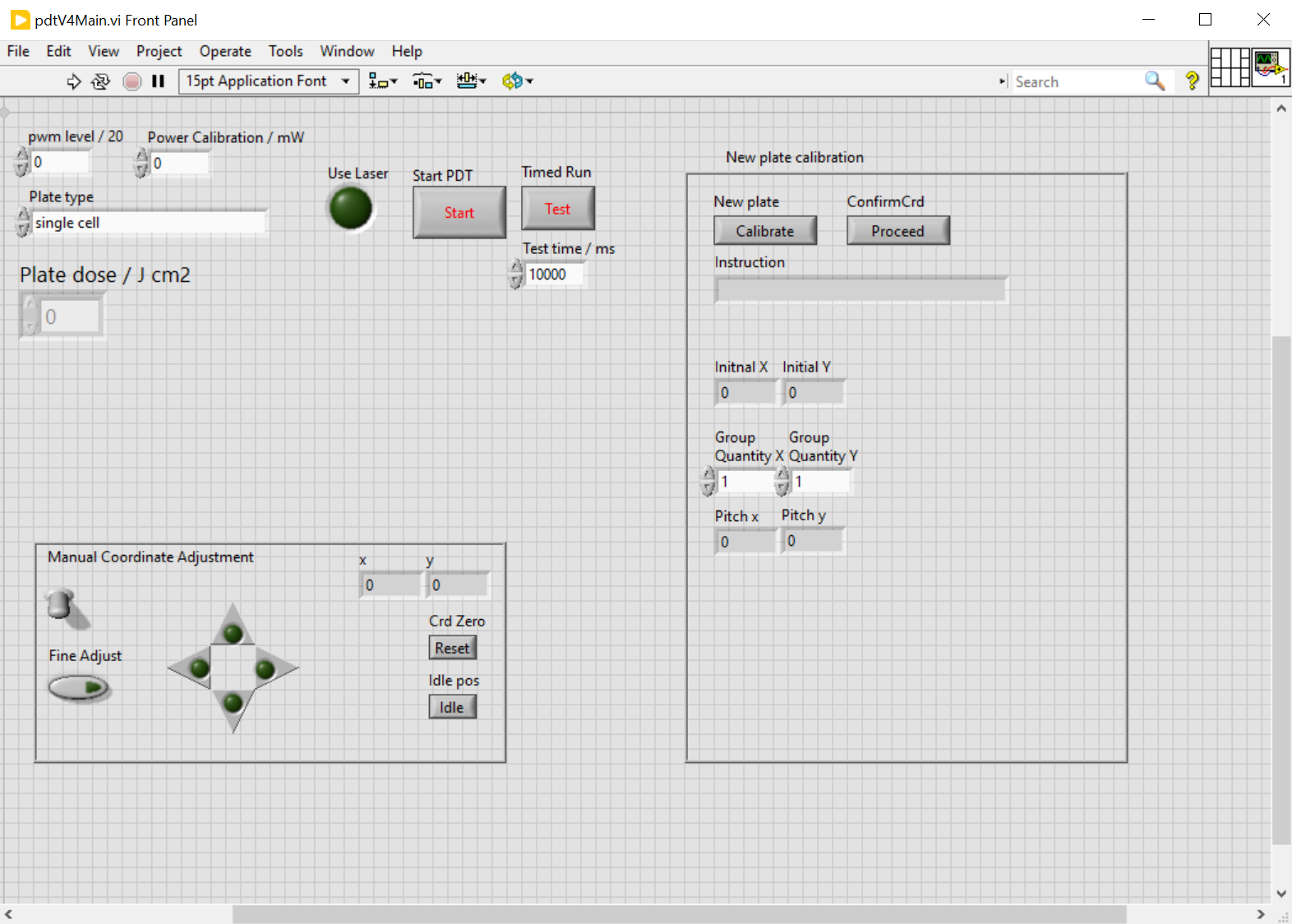
**

**The computer interface program.** This program controls the LED array and the robot arms of the illumination system. It also features the robot arm position calibration function. The program is constantly updating. The latest stable version is available at <https://github.com/springlabnu/pdtV4> as stated in the *Code Availability*.
